# Disparity of ovarian cancer survival between urban and rural settings

**DOI:** 10.1136/ijgc-2021-003096

**Published:** 2022-02-23

**Authors:** Keely Krolikowski Ulmer, Breanna Greteman, Nicholas Cardillo, Anthony Schneider, Megan McDonald, David Bender, Michael J Goodheart, Jesus Gonzalez Bosquet

**Affiliations:** 1 Gynecologic Oncology, The University of Iowa Hospitals and Clinics, Iowa City, Iowa, USA; 2 College of Epidemiology, The University of Iowa, Iowa City, Iowa, USA; 3 Department of Obstetrics and Gynecology, The University of Iowa Hospitals and Clinics, Iowa City, Iowa, USA

**Keywords:** ovarian cancer, gynecology, neoplasms, quality of life (PRO)/palliative care

## Abstract

**Objective:**

To determine if there is a difference in overall survival of patients with epithelial ovarian cancer in rural, urban, and metropolitan settings in the United States.

**Methods:**

We performed a retrospective cohort study using 2004–2016 National Cancer Database (NCDB) data including high and low grade, stage I-IV disease. Bivariate analyses used Student’s t-test for continuous variables and χ^2^ test for dichotomous variables. Kaplan-Meier curves estimated survival of patients based on location of residence, and univariate analyses using Cox proportional HR assessed survival based on baseline characteristics. Multivariate analysis was performed to account for significant covariates. Propensity score matching was used to validate the multivariate survival model. For all tests, p<0.05 was considered statistically significant.

**Results:**

A total of 111 627 patients were included with a mean age of 62.5 years for metroolitan (range 18–90), 64.0 years for rural (range 19–90) and 63.2 years for urban areas (range 18–90). Of all patients included, 94 290 were in a metropolitan area (counties >1 million population or 50 000–999 999), 15 386 were in an urban area (population of 10 000–49 999), and 1951 were in a rural area (non-metropolitan/non-core population). Univariate Cox proportional hazards models showed clinically significant differences in survival in patients from metropolitan, urban, and rural areas. Multivariate Cox proportional hazards models showed a clinically significant increase in HRs for patients in rural settings (HR 1.17; 95% CI 1.06 to 1.29). Increasing age and stage, non-insured status, non-white race, and comorbidity were also significant for poorer survival.

**Conclusion:**

Patients with ovarian cancer who live in rural settings with small populations and greater distance to tertiary care centers have poorer survival. These differences hold after controlling for stage, age, and other significant risk factors related to poorer outcomes. To improve clinical outcomes, we need further studies to identify which of these factors are actionable.

**HIGHLIGHTS**

- Patients with ovarian cancer living in rural areas have worse survival than those living in metropolitan areas
- Rural residency should be considered an independent risk factor for poorer ovarian cancer survival
- Further studies are needed to find actionable factors to improve disparities in survival of patients living in rural areas

## INTRODUCTION

Public health has addressed health inequalities for centuries, but the disparity in survival for 19th century urban residents due to overcrowding has now shifted to one for those living in rural areas.^1–3^ In the past several decades, the historical advantages of those living in rural areas has conversely become a ‘rural mortality penalty’ despite the all cause death rate in the United States reaching historic lows.^4^ Between 1999 and 2014, annual age-adjusted death rates and potentially excess deaths for the five leading causes of death in the United States (heart disease, cancer, unintentional injury, chronic lower respiratory disease, and stroke) were higher in rural areas.^5 6^ As rural America is home to 22.8% of US women aged 18 and older, addressing and examining the crisis of rural public health is of paramount importance.^7^

Patients living in rural areas have poorer cancer outcomes compared with similar populations in more metropolitan areas.^8–12^ Living in a rural area may be a contributing barrier to high-quality ovarian cancer care.^13–15^ A study done in 2021 concluded that rural residence and living long distances from the reporting hospital were associated with later-stage diagnoses and lower survival rates in young patients with cancer.^16^ Similarly, studies have suggested that patients with ovarian cancer living in rural areas present with later stage disease at diagnosis and have shorter survival than their urban counterparts.^17 18^ Over 99% of gynecological oncology providers in the United States work in metropolitan counties, despite approximately 20% of their patients living in rural counties. Studies examining risk factors for ovarian cancer have suggested that rurality is an independent risk factor in the United States and in other countries. These studies show possible, but inconclusive, statistical differences in ovarian cancer outcomes for patients living in rural areas, which prompts further investigation.^19–23^ Because of poorer survival, with nearly one in five of all patients with ovarian cancer living rurally, it is of utmost importance to examine the effect of rural residence on ovarian cancer outcomes.

We hypothesize that individuals with ovarian cancer living in rural areas have poorer outcomes than those living in urban and metropolitan areas. This study sought to assess the effect of rurality on clinical outcomes in patients with epithelial ovarian cancer from the National Cancer Database (NCDB).

## METHODS

### Study Population

**Figure 1:**
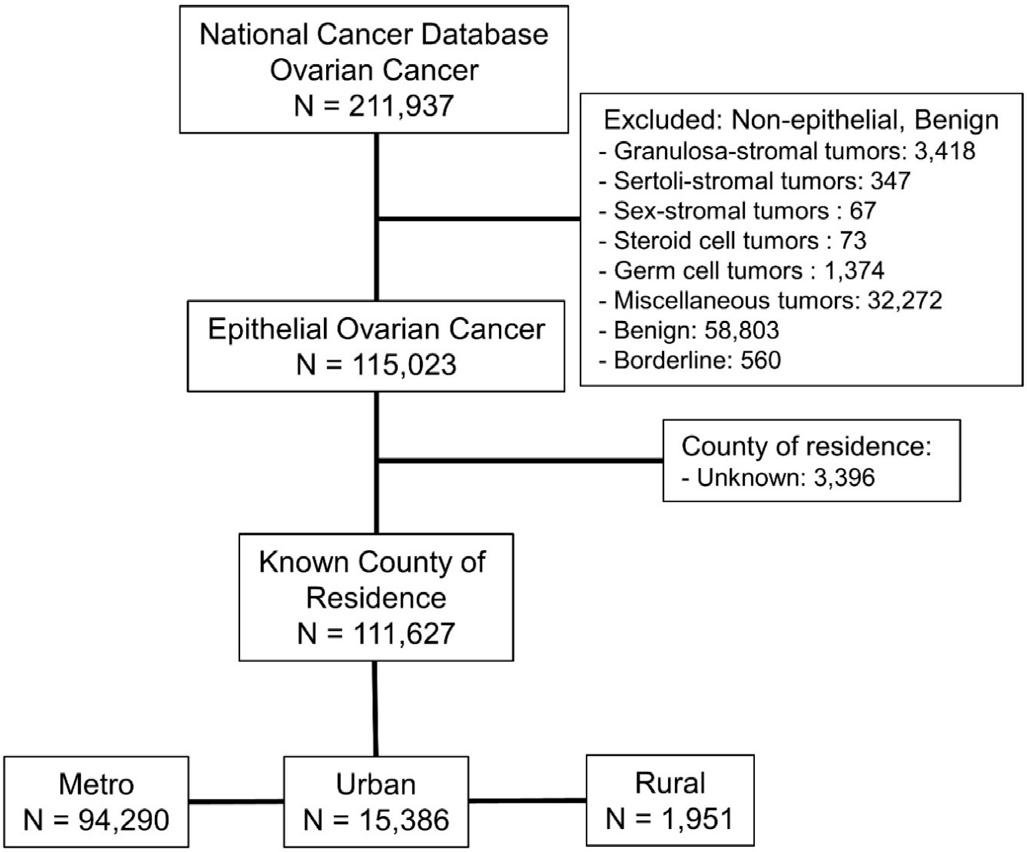
Inclusion and exclusion criteria from the National Cancer Database, ovarian cancer subset. Exclusion criteria were applied so that only those with epithelial ovarian cancer and known county of residence were included.

We performed a retrospective cohort study using NCDB data to identify women diagnosed with epithelial ovarian cancer between 2004 and 2016. Data were extracted from the ovarian cancer subset of the 2004–2016 NCDB, a nationwide oncology outcomes database for more than 1500 Commission on Cancer accredited cancer programs in the United States and Puerto Rico. Inclusion criteria were all patients with epithelial ovarian cancer and a known county of residence according to the NCDB database for the required years. We excluded patients with non-epithelial ovarian tumors or benign pathology, and those with an unknown county of residence (Figure 1).

Residence was defined using the National Center for Health Statistics urban–rural classification scheme for counties^24^ and divided into three categorical variables based on the census tract in the county where the patient lived at the time of diagnosis.^17^ Patients were categorized as metropolitan if they lived in a county of more than 50 000 people, urban if they lived in a county of 10 000–49 999, and rural if they lived in a county with less than 10 000 people.

### Statistical Analysis

Univariate patient characteristics included known determinants of health, including age, race, stage, location of residence, income, education, type of insurance, comorbidities, year of diagnosis, days to treatment and to surgery, distance to tertiary level hospital, and surgical outcomes. Distance to tertiary hospitals was measured from the centroid of the patient’s zip code (or the city if the zip code was not available) to the location of the hospital, based on the street address for the facility. Most variables were categorical: race, stage, location of residence, income, education, type of insurance, comorbidities, year of diagnosis, residual disease, and sequence of treatment. The rest were analyzed as continuous variables: age, days to treatment and to surgery, distance to tertiary level hospital. The categories of each variable are displayed in Table 1.

**Table 1:** Patients’ baseline characteristics based on their county of residence

	Total	Metropolitan	Rural	Urban	p-value
Total	111 627	94 290	1951	15 386	
Age (mean)	62.7	62.5	64	63.2	<0.001
Race					
White	96 697 (86.6%)	80 658 (85.5%)	1801 (92.3%)	14 238 (92.5%)	
American Indian	408 (0.4%)	261 (0.3%)	27 (1.4%)	120 (0.78%)	<0.001
Asian	3418 (3.1%)	3314 (3.5%)	4 (0.2%)	100 (0.65%)	<0.001
Black	8853 (7.9%)	8026 (8.5%)	105 (5.4%)	722 (4.7%)	<0.001
Pacific	213 (0.2%)	189 (0.2%)	1 (0.01%)	23 (0.16%)	0.04
Unknown	2038 (1.8%)	1842 (2.0%)	13 (0.7%)	183 (1.2%)	
Spanish					
Non-Spanish	99 475 (89.1%)	83 615 (88.7%)	1775 (91.0%)	14 085 (91.5%)	
Spanish	6271 (5.6%)	5941 (6.3%)	25 (1.3%)	305 (2.0%)	<0.001
Unknown	5881 (5.3%)	4734 (5.0%)	151 (7.7%)	996 (6.5%)	
Insurance					
Medicare	47 010 (43%)	38 721 (41.1%)	968 (49.6%)	7321 (47.6%)	
Medicaid	6785 (6.2%)	5672 (6.0%)	110 (5.6%)	1003 (6.5%)	0.01
Not insured	4503 (4.1%)	3722 (3.95%)	100 (5.1%)	681 (4.4%)	0.63
Other government	1041 (1%)	843 (0.9%)	20 (1.0%)	178 (1.15%)	0.25
Private	49 912 (45.7%)	43 365 (45.9%)	708 (36.3%)	5839 (37.9%)	<0.001
Insurance status unknown	2376 (2.2%)	1967 (2.1%)	45 (2.3%)	364 (2.4%)	0.60
Income ($)					
<38 000	17 405 (15.6%)	11 771 (12.5%)	788 (40.4%)	4846 (31.5%)	<0.001
38 000–47 999	25 369 (22.7%)	18 159 (19.3%)	743 (38.1%)	6467 (42.0%)	<0.001
48 000–62 999	30 105 (27%)	26 445 (28.0%)	351 (18.0%)	3309 (21.5%)	<0.001
≥63 000	38 548 (34.5%)	37 764 (40.1%)	58 (3.0%)	726 (4.7%)	
Unknown	200 (0.2%)	151 (0.2%)	11 (0.6%)	38 (0.2%)	
High school education (%)					
<7.0	28 828 (25.8%)	27 262 (28.9%)	200 (10.3%)	1366 (8.9%)	
7.0–12.9	37 225 (33.3%)	31 978 (33.9%)	531 (27.2%)	4716 (30.7%)	<0.001
13.0–20.9	27 835 (24.9%)	21 507 (22.8%)	633 (32.4%)	5695 (37.0%)	<0.001
≥21	17 590 (15.8%)	13 433 (14.2%)	578 (29.6%)	3579 (23.3%)	<0.001
Unknown	149 (0.1%)	110 (0.1%)	9 (0.5%)	30 (0.2%)	
Mean distance to care center (miles	35	27.4	88.4	72.9	<0.001
Charlson score	2.22	2.22	2.24	2.25	<0.001
Year of diagnosis					0.004
2004	5915 (5.3%)	4992 (5.3%)	108 (5.5%)	815 (5.3%)	
2005	6369 (5.7%)	5375 (5.7%)	103 (5.3%)	891 (5.8%)	
2006	6663 (6.0%)	5529 (5.9%)	112 (5.7%)	1022 (6.6%)	
2007	7385 (6.6%)	6267 (6.6%)	139 (7.1%)	979 (6.4%)	
2008	7910 (7.1%)	6613 (7.0%)	158 (8.1%)	1139 (7.4%)	
2009	8162 (7.3%)	6924 (7.3%)	158 (8.1%)	1080 (7.0%)	
2010	8336 (7.5%)	7045 (7.5%)	164 (8.4%)	1127 (7.3%)	
2011	8951 (8.0%)	7528 (8.0%)	153 (7.8%)	1270 (8.3%)	
2012	9430 (8.4%)	7980 (8.5%)	148 (7.6%)	1302 (8.5%)	
2013	10 340 (9.3%)	8698 (9.2%)	189 (9.7%)	1453 (9.4%)	
2014	10 627 (9.5%)	8969 (9.5%)	170 (8.7%)	1488 (9.7%)	
2015	11 154 (10.0%)	9538 (10.1%)	180 (9.2%)	1436 (9.3%)	
2016	10 385 (9.3%)	8832 (9.4%)	169 (8.7%)	1384 (9.0%)	
Grade					0.03
high	69 084 (61.9%)	58 132 (61.7%)	1203 (61.7%)	9749 (63.4%)	
low	11 028 (9.9%)	9370 (9.9%)	175 (9.0%)	1483 (9.6%)	
unknown	31 515 (28.2%)	26 788 (28.4%)	573 (29.4%)	4154 (27.0%)	
FIGO stage					
Stage I	24 327 (21.8%)	20 667 (21.9%)	417 (21.4%)	3243 (21.1%)	
Stage II	9965 (8.9%)	8475 (9.0%)	162 (8.3%)	1328 (8.6%)	0.83
Stage III	42 032 (37.7%)	35 155 (37.3%)	735 (37.7%)	6142 (39.9%)	<0.001
Stage IV	26 889 (24.1%)	22 855 (24.2%)	488 (25.0%)	3546 (23.0%)	0.89
Unknown	8414 (7.5%)	7138 (7.6%)	149 (7.6%)	1127 (7.3%)	
Days to treatment (mean)	11.3	11.3	10.9	11	0.14
Days to surgery (mean)	28.4	28.3	28.3	29.9	0.27
Residual disease after surgery					
R0	47 667 (42.7%)	40 383 (42.8%)	769 (39.4%)	6515 (42.3%)	<0.001
R1	31 984 (28.7%)	26 654 (28.3%)	640 (32.8%)	4690 (30.5%)	
Unknown	31 976 (28.6%)	27 253 (28.9%)	542 (27.8%)	4181 (27.2%)	
Sequence of treatment					
Primary cytoreduction	47 661 (42.7%)	40 250 (42.7%)	804 (41.2%)	6607 (42.9%)	0.03
Neoadjuvant chemotherapy	13 127 (11.8%)	10 985 (11.7%)	247 (12.7%)	1895 (12.3%)	
Unknown	50 839 (45.5%)	43 055 (45.7%)	900 (46.1%)	6884 (44.7%)	

Baseline characteristics of the study patient included age, race, Spanish speaking, insurance, income, high school education, distance to care center, Charlson comorbidity score, year of diagnosis, grade, FIGO stage, days to treatment, days to surgery, residual disease, and sequence of treatment. Mean value of continuous features are displayed: age, days to treatment and to surgery, distance to tertiary level hospital. Data are numbers (%) unless stated otherwise.

FIGO, International Federation of Gynecology and Obstetrics.

A crude comparison of residence and survival was conducted with Kaplan-Meier curves. We performed univariate analyses using the Cox proportional hazards models to assess survival of patients based on baseline characteristics. Cox proportional hazards models were created for variables significant (p<0.05) in univariate analyses. Univariate survival analysis was performed with all initial continuous and categorical categories as described previously.

For the multivariate analysis we created dummy variables for three of the categorical features to simplify analysis and interpretation of the data: race was dichotomized into white and non-white, and Charlson comorbidities score into ≤2 and >2, while insurance status was categorized as government (referent), private, and non-insured. Then multivariate Cox proportional hazards models were created to adjust for all significant covariates associated with survival. Furthermore, to account for any assignment and/or selection biases associated with referral centers, we performed a propensity score matching for the outcome of interest and covariates included in the Cox model. Nearest neighbor matching was used for propensity matching with the *MatchIt* R package.^25–27^ After matching, 41 413 people in each survival group were available for analysis. To visualize multivariate survival model results, we created forest plots (Online supplemental file 1). For all tests, p values less than 0.05 were considered statistically significant. Data analysis was performed using R environment for statistical computing and graphics (www.r-project.org). In accordance with the journal’s guidelines, we will provide our data for the reproducibility of this study in other centers if requested.

10.1136/ijgc-2021-003096.supp1 Supplementary data

## RESULTS

A total of 111 627 patients were included in our analysis, with 84.5% from metropolitan areas, 13.8% from urban areas, and 1.7% from rural areas. Patient demographics are depicted in Table 1. Patients from rural patients (1.7%) were older (64 years; p<0.001), more often white (92.3%; p<0.001), poor (40.4% with income <$38 000 per year; p<0.001), uneducated, and were diagnosed with later stage cancers (27.1% stage IV; p<0.001) (Table 1).

### Survival Analysis

**Figure 2:**
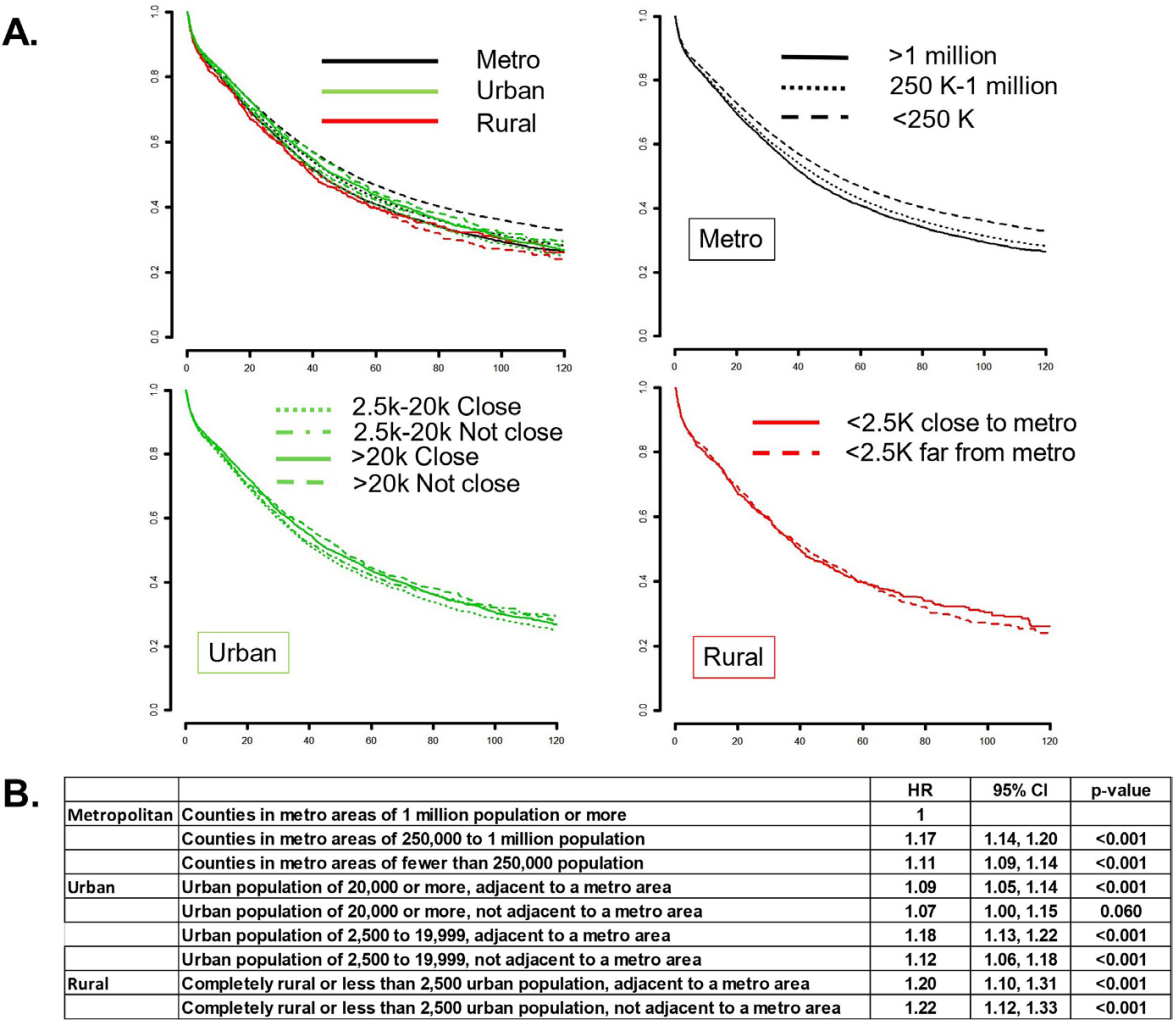
Univariate survival analysis. (A) Kaplan-Meier survival curves based on location of residence defined by the state and county FIPS code of the patient recorded at the time of diagnosis against 2013 files published by the United States Department of Agriculture Economic Research Service (http://www.ers.usda.gov/dataproducts/rural-urban-continuum-codes). Upper left: comparing survival of ovarian cancer patient by metropolitan, urban or rural site of residence; upper right: comparing ovarian cancer survival based on metropolitan areas of residence; lower left: comparing ovarian cancer survival based on urban areas of residence; lower right: comparing ovarian cancer survival based on rural areas of residence. (B) Results of the univariate analysis of survival showing HRs, 95% CIs, and p-values of patients living in metropolitan, urban, and rural counties.

In univariate analyses, patients with increasing age (HR 1.03, 95% CI 1.03 to 1.03), non-white race (1.18, 1.15 to 1.22), increased Charlson comorbidity index (1.16, 1.14 to 1.18), lack of insurance (1.24, 1.18 to 1.31), income <$38 000 (1.13, 1.10 to 1.15), increasing FIGO (International Federation of Gynecology and Obstetrics) stage (stage II: 2.00, 1.90 to 2.10; stage III: 4.55, 4.39 to 4.72; stage IV: 8.34, 8.03 to 8.66), and county of residence (rural (<2500 population), not close to a metropolitan area: 1.17, 1.06 to 1.29) had lower survival. Univariate results also revealed significant differences in ovarian cancer outcomes for those living in rural areas compared with those in metropolitan areas (with population of 1 million or more). Patients with ovarian cancer living in rural areas were found to have worse survival than patients living in urban and metropolitan areas (HR 1.20, 95% CI 1.10 to 1.31), or 20% increased risk of dying from disease when the rural county was located adjacent to a metropolitan area, and 1.22 (p<0.001) in rural regions not adjacent to a metropolitan area (Figure 2).

**Figure 3:**
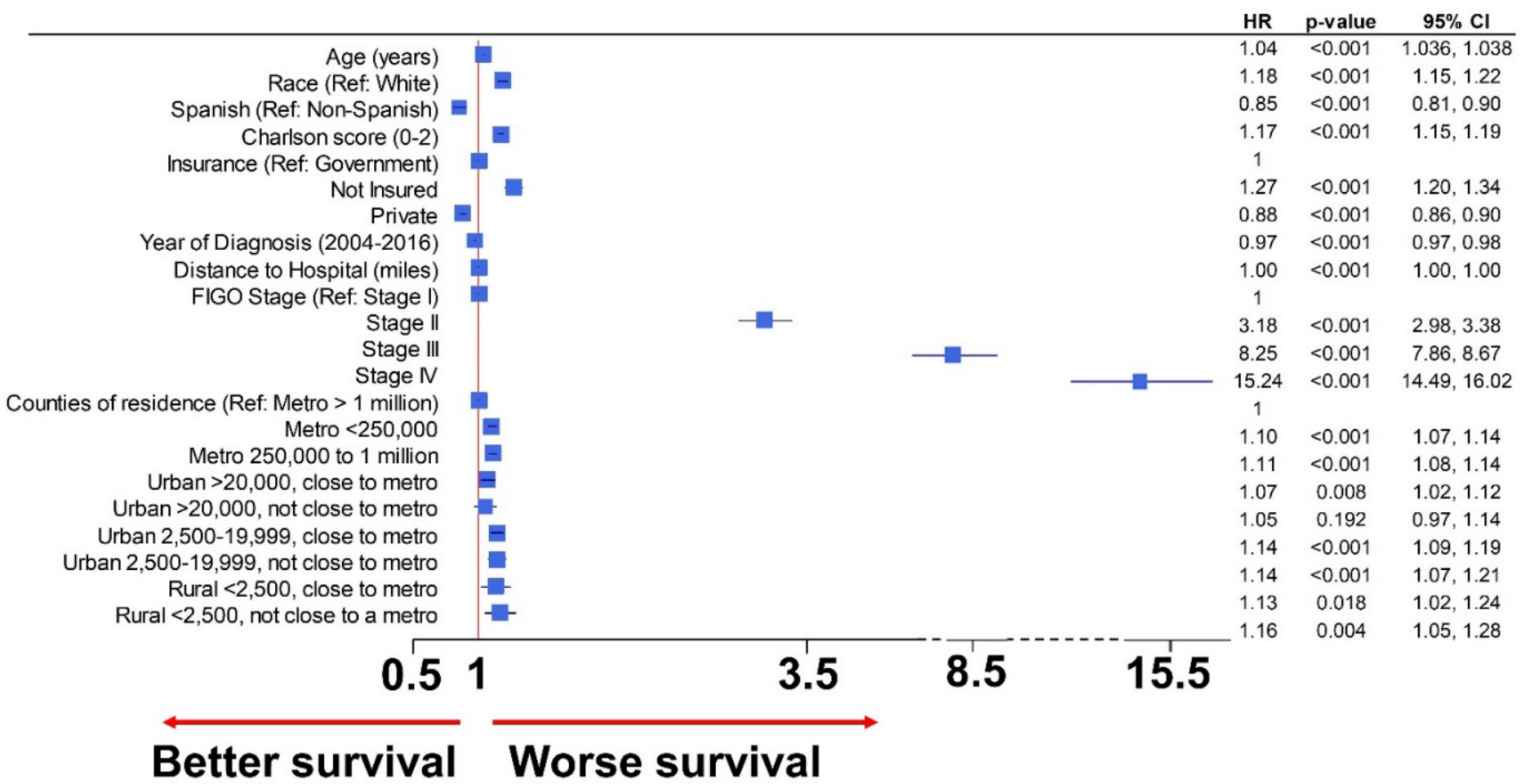
Multivariate survival model after propensity matching for significant covariates. Forest plot of significant variables after propensity score matching of significant covariates in the multivariate Cox model. Patients with ovarian cancer that live in rural counties had poorer survival than those living in metropolitan counties (HR 1.16, 95% CI 1.05 to 1.28), even after propensity score matching and accounting for age, stage, race, Spanish background, type of insurance, year of diagnosis, and distance to care center. FIGO, International Federation of Gynecology and Obstetrics.

In the multivariate Cox proportional HR, patients living in the smallest counties had an increased hazard for mortality (HR 1.17, 95% CI 1.06 to 1.29, p=0.002) after controlling for age, race, Spanish background, Charlson comorbidity score, insurance, year of diagnosis, FIGO stage, and distance to hospital. Patients with ovarian cancer living in rural areas had worse survival after propensity score matching and accounting for all significant covariates (Figure 3). Furthermore, all patients with ovarian cancer living in counties with a population less than 20 000 (urban and rural) had worse outcomes than those living in metropolitan areas of more than one million inhabitants. A forest plot with independently significant variables for survival in the multivariate model after propensity score matching is represented in Figure 3.

## DISCUSSION

### Summary of Main Results

In this review of registry data using the NCDB data from 2004 to 2016 of patients with epithelial ovarian cancer, patients living in rural areas had worse overall survival outcomes than those living in metropolitan areas. These results hold true after multivariate survival analysis and propensity score matching. Of note, most significant variables in the univariate analysis, later introduced in the multivariate model, have previously been reported to influence survival.^28^ This suggests that rurality is an independent risk factor for poor outcomes in patients with ovarian cancer.

### Results in the Context of Published Literature

Rurality as an independent risk factor has not previously been reported for ovarian cancer. It has been suggested that rurality can influence the outcome in patients with ovarian cancer, but rurality as an independent outcome variable for survival has not been reported.^9 13–17^ In our study, we demonstrated rural residence as an independent risk factor. However, in our multivariate analysis, age, insurance, comorbidity, and stage were also significant. These significant variables are more prevalent in rural populations.^12 29 30^ These findings display the compounding effect of comorbidity, later stage at diagnosis, distance to healthcare centers/specialty care physicians, lack of insurance, and socioeconomic status, which we believe may together represent the disparity of rurality. To provide for rural American women (22.8% of women aged 18 years and older),^7^ providers must recognize that rurality alone is a complex risk factor that leads to poorer outcomes in patients with ovarian cancer.

Our findings are consistent with other studies which show that the patterns of care associated with rural residence vary by cancer type, stage at diagnosis, and geographical residence.^9^ In regards to other similar gynecological studies, one study in Kentucky examining patients living in rural areas diagnosed with endometrial cancer demonstrated that rural women more often lacked commercial insurance, underwent less comprehensive surgical evaluation, and had less multimodality treatment.^31^ During a study period between 2008 and 2010, rural women aged 18–64 years reported the highest rates of delayed care or no medical care due to cost (18.6%) and no health insurance coverage (23.1%), with both rates increasing since 2002–2004.^7 32^ Distance to specialty care providers is likely to contribute to the barriers for women living in rural areas. Healthcare providers, especially those at tertiary hospitals, can coordinate more comprehensive treatments, including surgery, radiation, or chemotherapy.

### Strengths and Weaknesses

The strengths of our study are that it is a large, population-based study including all patients with epithelial ovarian cancer on the NCDB, which is about 75% of all cases in the USA.^29^ Also, we controlled for covariates known to affect overall survival, including age, race, and stage. Furthermore, to validate results and decrease possible biases, we repeated the analysis after propensity score matching. Our study has some limitations inherent to the nature of the design (retrospective) and because some data are not collected by the NCDB, like disease recurrence. Recurrence of disease is notably unreliable as an outcome variable but endorsed as an important determinant. The diagnosis of recurrence depends on multiple factors, including how it is defined,^33 34^ making it difficult to quantify and control. Unfortunately, the NCDB started to collect data about location of treatment in 2014, and only recorded whether treatment was performed at the reporting facility or elsewhere. We should not assume that patients received their surgery and/or chemotherapy in the reporting cancer center because they were referred to them. Some patients may have received only part of their treatment in the referred center. We are working on state-wide specific data that will better explain some of these issues.

### Implications for Practice and Future Research

Addressing this rural disparity is a complex issue. Some factors that may contribute to this disparity in ovarian cancer are lack of specialty care physicians, including gynecological oncologists, but in a more important sense, the services of general practice obstetrician gynecologists who are familiar with women’s health. According to a survey performed by the American College of Obstetricians and Gynecologists in 2008, only 6.4% of obstetrician gynecologists practiced in rural settings and by 2010 49% of US counties accounting for 10.1 million women lacked an obstetrician gynecologist.^7^ This shortage of rural obstetrician gynecologist care is complicated by the closure of many labor and delivery units, causing even fewer rural recruitment capabilities for smaller rural hospitals. Potential areas for improvement could therefore include initiatives to promote recruitment of gynecology-trained physicians to these rural areas and to raise awareness among primary care physicians about women’s healthcare screening guidelines, and more specifically, signs and symptoms of ovarian cancer. Another potential improvement could include increased access or ease of arranging affordable transportation alternatives for patients of lower socioeconomic status living in rural areas because distance to care was found to be significant. Increased outreach clinic access for rural populations may build on the better outcomes demonstrated for chemotherapy care by gynecological oncologists.^35^

### Conclusions

This study shows that women with ovarian cancer living in rural areas are at significant risk of worse overall outcomes, and rurality is an independent risk factor. Other factors may also influence survival outcomes, such as distance to care centers, insurance status, race, and background. It is important to recognize that disparity in care of patients with ovarian cancer in rural settings is an important and complex issue that deserves further study and must be recognized when caring for these patients.

**Table 2:** Survival multivariate Cox proportional HR analysis

	HR	p-value	95% CI
Age (years)	1.03	<0.001	1.03 to 1.03
Race (reference: white)	1.18	<0.001	1.15 to 1.22
Spanish (reference: non-Spanish)	0.89	<0.001	0.85 to 0.93
Charlson score (0–2)	1.16	<0.001	1.14 to 1.18
Insurance (reference: government)	1		
Not insured	1.24	<0.001	1.18 to 1.31
Private	0.87	<0.001	0.84 to 0.89
Year of diagnosis (2004–2016)	0.99	<0.001	0.98 to 0.99
Distance to hospital (miles)	1.00	<0.001	1.00 to 1.00
FIGO stage (reference: stage I)	1		
Stage II	2.00	<0.001	1.90 to 2.10
Stage III	4.55	<0.001	4.39 to 4.72
Stage IV	8.34	<0.001	8.03 to 8.66
Counties of residence (reference: metropolitan>1 million)			
Metropolitan<2 50 000	1.11	<0.001	1.08 to 1.15
Metropolitan 250 000 to 1 million	1.11	<0.001	1.08 to 1.13
Urban>20 000, close to metro	1.05	0.03	1.01 to 1.10
Urban>20 000, not close to metro	1.04	0.34	0.96 to 1.12
Urban 2500–19 999, close to metro	1.13	<0.001	1.08 to 1.18
Urban 2500–19 999, not close to metro	1.13	<0.001	1.06 to 1.20
Rural <2500, close to metro	1.12	0.02	1.02 to 1.24
Rural <2500, not close to a metro	1.17	0.002	1.06 to 1.29

HR, p-value and 95% CI for variables shown to be of significance in the univariate analysis with significant difference shown in those of rural populations. For the multivariate model we created dummy variables for three of the categorical variables: race was dichotomized into white and non-white, and Charlson comorbidities score into ≤2 and >2, while insurance status was divided into government (reference), private, and non-insured.

FIGO, International Federation of Gynecology and Obstetrics.

## Data Availability

All data relevant to the study are included in the article or uploaded as supplementary information.
