# Supplementary material for: Disparity of ovarian cancer survival between urban and rural settings
Source: Int J Gynecol Cancer. 2022 Feb 23;32(4):540–6. doi: 10.1136/ijgc-2021-003096 (PMC8995817; doi:10.1136/ijgc-2021-003096)

Supplementary Figure S1. ***Forest plot of multivariate analysis of survival.*** Forest plot demonstrating survival outcome of significant variables determined by the multivariate analysis. Squares represent the HR and the lines their 95% CI. Urban and rural site of residence had worse survival for ovarian cancer patients.

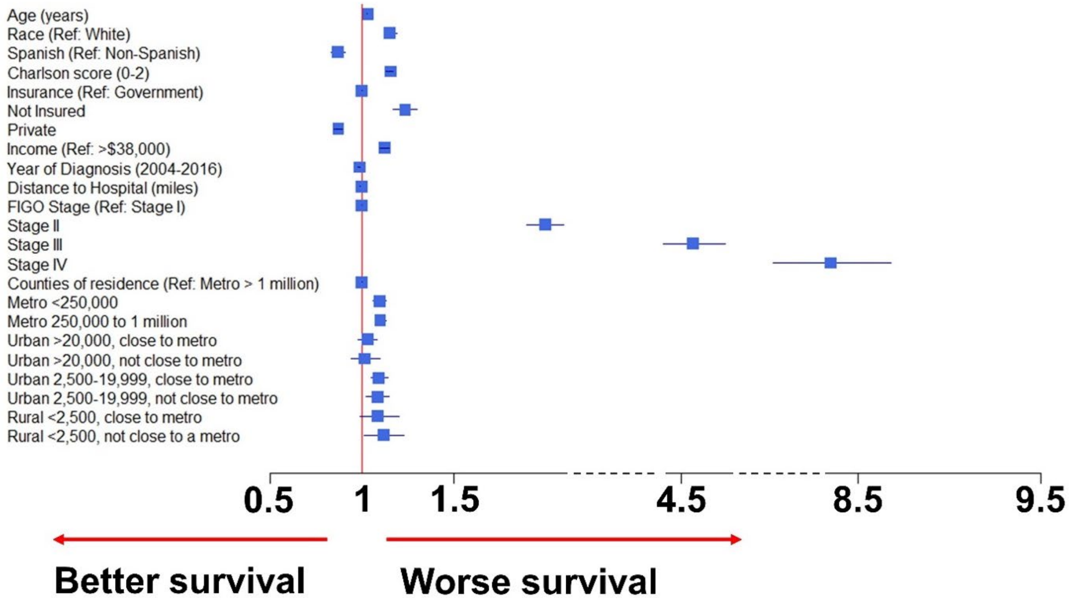

Supplement: Supplementary data [file ijgc-2021-003096supp001.pdf]
